# Effects of a Mental Health Intervention in Athletes: Applying Self-Determination Theory

**DOI:** 10.3389/fpsyg.2019.01875

**Published:** 2019-08-13

**Authors:** Stephen Shannon, Donncha Hanna, Tandy Haughey, Gerard Leavey, Conor McGeown, Gavin Breslin

**Affiliations:** ^1^Sport and Exercise Sciences Research Institute, Ulster University, Newtownabbey, United Kingdom; ^2^Institute of Mental Health Sciences, Ulster University, Derry, United Kingdom; ^3^School of Psychology, Queen’s University Belfast, Belfast, United Kingdom; ^4^Centre for Evidence and Social Innovation, Queen’s University Belfast, Belfast, United Kingdom; ^5^Bamford Centre for Mental Health and Wellbeing, School of Psychology, Ulster University, Coleraine, United Kingdom

**Keywords:** health psychology, sport, mediation, well-being, needs satisfaction

## Abstract

**Introduction:** Many sport associations have responded to mental health issues in sport through the inclusion of self-management programs, such as mindfulness training, which may improve well-being through increasing one’s competence in self-regulating stressors. Yet, the mechanisms accounting such changes lack a theoretical basis, particularly in athletes.

**Aim:** To determine the effect of a mental health intervention comprising a mindfulness program for promoting well-being, reducing stress, and increasing competence in mental health self-management. This is the first study among athletes to test the mechanisms of change in a mindfulness program using Self-Determination Theory (SDT).

**Methods:** A 2 (groups) × 2 (time-point) non-randomized controlled trial was conducted, and between-groups baseline differences were firstly assessed. Two competing regression models assessing singular and serial indirect mediating mechanisms were conducted, in which mindfulness (Model 1) and competence satisfaction (Model 2) were both tested as primary and secondary mediators predicting change scores in stress and well-being. Demographic variables (i.e., gender, age) were controlled for in the analyses.

**Results:** Two hundred and thirty-eight student athletes (mean age = 20.47 years, *SD* = 3.30, 57.6% = males) participated, with 108 in the intervention group who received an instructional workshop, and a home-directed mindfulness program comprising daily meditation sessions. No baseline differences were found between intervention and control groups. In Model 1, mindfulness was not directly enhanced by the intervention, subsequently resulting in no indirect effects on competence, stress and well-being. In Model 2, the intervention was directly related to positive changes in competence (β = 0.39, *p* < 0.05), subsequently resulting in indirect effects on mindfulness awareness (β = 0.07, *p* < 0.05), stress (β = −0.06, *p* < 0.05), and well-being (β = 0.05, *p* < 0.05). In addition, serial indirect effects for the intervention on stress were present through competence and mindfulness awareness in sequence (β = −0.02, *p* < 0.05), and; on well-being through competence, mindfulness awareness, and stress in sequence (β = 0.01, *p* < 0.05; *R*^2^ = 0.54).

**Conclusion:** Mindfulness-based mental health interventions may be effective at reducing stress and promoting well-being in athletes, with the caveat that attention is given to the inclusion of mental health competence promotion in program design. However, it remains unclear whether increasing mindfulness itself can exert additional salutary effects. Our findings have an important bearing on how mindfulness programs are developed within athlete mental health interventions.

## Introduction

Well-being is defined as a state of optimal functioning (Ryan and Deci, 2017) and a key component of a two-continua model of mental health (Keyes, 2005). As a theoretical construct, well-being is characterized by psychological (i.e., a sense of purpose, realizing one’s potential), emotional (i.e., positive affective states, reduced negative affect) and social (i.e., relationships) dimensions (Keyes, 2005). Stress, inversely related to well-being (Keyes, 2005; Huppert, 2009; Diener et al., 2018), occurs when one feels overwhelmed or unable to cope as a result of pressures (Mental Health Foundation, 2018), and hence requires a preventative and treatment response. Student-athletes (or collegiate athletes) are prone to stress because of co-existing academic, social and sporting demands (Wilson and Pritchard, 2005; Bennett, 2007). For example, student-athletes report pressure to achieve in both academic and sporting pursuits, a constrained social life, relationship difficulties and examination pressures (Gavrilova et al., 2017). Student-athletes have higher clinical and sub-clinical risks for behavioral mental health problems (e.g., substance misuse, eating disorders, gambling) compared to non-athletes (Moreland et al., 2018). Moreover, student-athletes are at least as, or more likely, to experience mood disorders compared to non-athletes (Donohue et al., 2018). Furthermore, due to the physical, and often aggressive nature of sport, student-athletes can incur physical injury, and experience emotional and physical fatigue from competition and over-training (Putukian, 2016). Athletes may also experience performance pressures from coaches, teammates and spectators, and often strive to succeed at the expense of personal well-being (Abedalhafiz et al., 2010; Breslin et al., 2018b). When left untreated, such stressors can manifest in impaired functioning (Moreland et al., 2018), highlighting the need for mental health self-management interventions.

Mental health self-management refers to monitoring how one’s mental health is impacting upon daily functioning, and utilization of strategies that protect and promote mental health (Wolf, 1996). Many student-athletes report that they do not have the skills, or resources, to self-manage mental health, resulting in maladaptive coping strategies (e.g., substance misuse) (Eisenberg et al., 2007; Hunt and Eisenberg, 2010). Mindfulness is an example of a self-management strategy available to athletes (Noetel et al., 2017). Mindfulness is defined as a mental state characterized by an awareness of present events and experiences (Brown and Ryan, 2003), achieved mainly through meditative practices (Sappington and Longshore, 2015). Although mindfulness has traditionally been guided by practitioners in group-based or individual therapies (Langley, 2013), recently, mindfulness programs have become widely available through auditory meditative guidance in smartphone applications (Howells et al., 2016).

In sport, most mindfulness interventions intend to improve performance-related outcomes (e.g., improving psychological flow during performance) rather than mental health (Sappington and Longshore, 2015; Noetel et al., 2017). While mindfulness intervention studies for improving mental health outcomes among athletes are promising (e.g., Vidic et al., 2017; Glass et al., 2018), so far, none have examined the theoretical mechanisms of change that may explain the benefits experienced. To ascertain how changes occur during mindfulness programs, theoretical constructs are modeled to assess the indirect effect of a treatment (X) on an outcome (Y) through one or more mediators (M) (Kok et al., 2004).

Relevant to the monitoring and ability components of the mental health self-management construct (Wolf, 1996), it is proposed that when one perceives mental health competence, one can cope adaptively, regulate stress and experience a positive sense of well-being (Gustafsson and Skoog, 2012). Self-Determination Theory (SDT) (Ryan and Deci, 2000) posits that competence, an innate psychological need and feeling a sense of effectiveness in one’s environment, is essential for optimal well-being. Of the three core psychological needs in SDT (i.e., competence, autonomy, relatedness) competence has been shown to have clear theoretical links with self-management, and indeed, a comprehensive body of research indicates that competence satisfaction is robustly related to positive mental health (Ryan and Deci, 2017). Models of SDT (Vallerand, 1997) outline that psychological needs exist and influence each other at three levels, i.e., situational (here and now), contextual (specific domains) and global (day-to-day). At the contextual level of mental health, researchers have shown that competence is linked to enhanced well-being (Mikolajczak et al., 2015) and reduced stress (Jex et al., 2001). Moreover, validated health domain measures of competence have been developed from a SDT perspective (Williams and Deci, 1996). Hence, given the clear theoretical links, mental health competence can be operationalized in a self-management intervention aiming to promote well-being.

In SDT, Ryan and Deci (2000) outline that needs-support (i.e., provision of choice, positive feedback, and caring dialogue) from intervention instructors has important implications for participants’ needs satisfaction, which ultimately aides in the initiation of health behavior change (e.g., mindfulness practices, exercise) and well-being. Indeed, from an interpersonal perspective, health interventions delivered in a needs-supportive environment have been shown to improve participants’ perceived autonomy-support, which subsequently resulted in improved needs satisfaction and well-being (Shannon et al., 2018). However, beyond such social-contextual factors, individuals can also draw upon internal psychological skills processes to satisfy their needs and well-being, such as one’s ability to be mindful of present events and experiences (Weinstein and Ryan, 2011; Ryan and Deci, 2017). In other words, through having an improved awareness and attention of the present moment, a person can reflectively self-manage the thoughts, and ultimately regulate feelings and basic needs satisfaction (Schultz and Ryan, 2015).

While SDT research on interpersonal predictors of needs satisfaction is extensive (Ryan and Deci, 2017), a small but growing number of studies show that mindfulness is related to competence satisfaction, and consequent mental health outcomes (Chang et al., 2018). In a temporal sense, it has been proposed that competence satisfaction is a corollary of mindfulness, such that mindful states provide individuals with a greater awareness of ongoing events, and subsequent purposive selection of need-satisfying experiences (Campbell et al., 2016; Campbell et al., 2017). In support of this hypothesis, correlational studies (Schultz et al., 2015; Chang et al., 2018) have shown that mindfulness is positively related to competence satisfaction which indirectly predicted stress reductions and improvements in well-being. However, it has also been shown that competence satisfaction is a precursor to mindfulness, and predicted improvements in employee well-being through mindfulness (Olafsen, 2017). With this view, it is proposed that competence satisfaction can be thought of as a resource that enables a person to be mindful, which therein provides individuals with an awareness that supports positive psychological well-being. Indeed, Brown et al. (2007) have emphasized that most research has taken the perspective of mindfulness as a facilitative factor of needs satisfaction, yet it is equally probable that psychological needs satisfaction cultivates mindful states.

Therefore, the temporal nature of the competence–mindfulness relationship remains unclear and requires further theoretical assessment (Brown et al., 2007; Creswell, 2017), and has yet to be rigorously assessed through an intervention study using SDT. Testing these questions has important theoretical and practical implications for the way in which mental health interventions with a mindfulness component are designed. Hence, the inclusion of SDT constructs in the analyses of a mindfulness intervention are warranted to contribute to current theoretical understanding of the mechanisms of change in mindfulness interventions. As such, the aim of this study was to determine whether a mental health intervention could improve well-being through reducing stress, and enhancing mindfulness and mental health competence.

### Study Hypotheses and Models Tested

**FIGURE 1:**
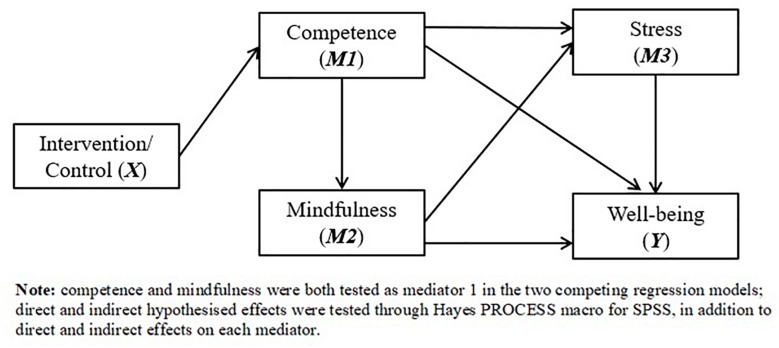
Hypothesized competing regression models assessing the effect of a mental health intervention (*X*) on well-being (*Y*), through mental health competence/mindfulness (*M1*), mental health competence/mindfulness (*M2*) and stress (*M3*).

The intervention was analyzed through two competing regression models comprising theoretically driven hypotheses. In both Models 1 and 2, well-being was designated as the dependent variable (*Y*), with participation in the intervention as the independent variable (*X*). To test the temporal relationship between competence satisfaction and mindfulness, in Model 1, mindfulness was designated as the primary mediator (*M1*), competence satisfaction (i.e., in self-managing mental health) was designated as mediator 2 (*M2*), and stress as mediator 3 (*M3*) (see Figure 1). The intervention was hypothesized to directly increase mindfulness (Hypothesis 1; H1), was in turn hypothesized to mediate the effects of the intervention on competence satisfaction (Hypothesis 2; H2). Considering stress has been inversely related to both mindfulness and competence, the intervention was hypothesized to indirectly effect stress through mindfulness (Hypothesis 3; H3), and through mindfulness and competence in sequence (Hypothesis 4; H4). Lastly, the intervention’s effects on well-being were hypothesized to be indirectly influenced through a combination of singular (i.e., intervention > mindfulness > well-being), double (i.e., intervention > mindfulness > competence > well-being; intervention > mindfulness > stress > well-being), and triple (i.e., intervention > mindfulness > competence > stress > well-being) sequential mediating pathways (Hypothesis 5; H5). In Model 2, competence satisfaction was designated the primary mediator (*M1*), while mindfulness was designated as mediator 2 (*M2*), and stress as mediator 3 (*M3*). We explored all of the above hypotheses, assuming the same direction of relationships.

## Materials and Methods

### Design, Inclusion Criteria, Recruitment Setting and Procedure

Ethical approval was granted by Ulster University (January 2017). All participants provided informed consent prior to their involvement. A mixed 2 (groups) × 2 (time-points) non-randomized controlled trial was conducted and reported using the Transparent Reporting of Evaluations with Non-randomized Designs (TREND) statement (Des Jarlais et al., 2004). It was not possible to implement a waiting-list controlled randomized design due to several foreseen practical considerations. These included; limited human resources to deliver the workshop across multiple university courses, and; student-athletes’ unavailability beyond the specified study time period because of travel, academic and work commitments. However, efforts were made to reduce the potential for contamination, as outlined below.

Inclusion criteria was based on participants responding “yes” to the following survey question consistent with the definition of sport, “are you an athlete involved in a structured, competitive physical activity?” (Rejeski and Brawley, 1988), resulting in 238 in the final sample, and exclusion of 58 non-athletes. Intervention participants were recruited by a verbal presentations which supplemented content from sport and exercise psychology modules in three academic sport courses. No academic course credit was received for engagement with the intervention, and participation was voluntary. Control participants were recruited by the research team through a range of sport centers and sports clubs, and selected university courses that did not comprise intervention participants. From March to April 2018, trained researchers led survey data collection under quiet classroom conditions, and participants completed the survey through online computer devices at baseline (Time 1), and two-weeks following the intervention (Time 2). The survey included descriptive items on the participants’ gender, sport, and age.

### Intervention

The State of Mind Ireland (Lawlor et al., 2015; Breslin et al., 2018a, 2019) intervention is a mental health awareness intervention, comprising an instructional workshop on mental health and mindfulness, and home-directed mindfulness training program. The intervention workshop took place in a seminar classroom on university campuses. Workshops lasted approximately 90 min (see Table 2), wherein each workshop comprised an average of 49 participants. The SOMI program was delivered by a psychiatrist and a student counselor with extensive course delivery experience. To ensure further available mental health support, the intervention deliverers consistently signposted participants to freely available clinical help at the university.

The workshop content was designed around SDT principles (Stone et al., 2009), to the extent that the activities and tutor delivery-style were provided through a needs-supportive environment^1^ that acknowledged participant input through open-ended questions, included regular positive instructional feedback, and empathetic and caring communication (e.g., use of the word “may” instead of “should” when providing instructions). For instance, the workshop introduced mental health as a positive concept, and asked participants to reflect on common stressors, and discuss their knowledge of mindfulness as a mental health self-management tool. Participants viewed vignettes of prominent athlete meditators, and then feedback on how a mindful state may improve participants’ competence to manage stressors, and promote mental health both in sport and university life contexts.

The second half of the workshop comprised instructions on the mindfulness mobile application that was designed by an online healthcare company specializing in meditation. The application included fourteen daily sessions comprising auditory and visual guidance, such as mindful body scanning for physical sensations, counting inhalations and exhalations, and noting thoughts and feelings. As part of the workshop participants engaged in a one minute-long guided taster session. Using needs-supportive communication, the workshop deliverers encouraged the participants to complete the daily sessions as much as possible during the a two-week period, with the application allowing for self-selection of the session durations (i.e., 5, 10, 15 or 20 min in length). Further positive instructional reminders were sent to participants through email and SMS acknowledging the challenges of mindfulness training, and encouragement to continue with the program. To assess adherence to the sessions, at follow-up the intervention participants reported on how many mindfulness sessions they completed by answering a single questionnaire item ranging from none through to 14.

### Outcomes

#### Mindfulness

Mindfulness was measured using Brown and Ryan (2003) Mindfulness Attention Awareness Scale (MAAS), a 15-item questionnaire designed to assess attention to, and awareness of, day-to-day experiences. All items were scored on a 6-point Likert scale ranging from “almost always” (1), to “almost never” (6), with higher scores reflecting better mindfulness. The MAAS is a valid and reliable measure, with several studies showing a unidimensional factor structure (Brown and Ryan, 2003; MacKillop and Anderson, 2007) and a Cronbach’s alpha = 0.88. An example item from the MAAS is: “I could be experiencing some emotion and not be conscious of it until some time later.”

#### Competence Satisfaction

The Perceived Competence Scale (PCS; Williams and Deci, 1996) was adapted and used to measure the participants’ competence with respect to self-managing mental health. All four items were scored on a 7-point Likert scale, with higher scores indicating higher competence. An example item includes: “I feel confident in my ability to manage my mental health.” The PCS is a valid and reliable tool for measuring domain-level competence (Williams et al., 1998), with a Cronbach’s alpha = 0.94 within the present study.

#### Stress

Stress was measured using the 10-item Perceived Stress Scale (PSS) (Cohen et al., 1994). The PSS assesses the appraisal of stress in day-to-day experiences and demonstrates excellent psychometric properties, with a unidimensional structure (Roberti et al., 2006; Lee, 2012). Each item assessed on a 5-point Likert scale ranging from 0 (“never”) to 4 (“very often”), with lower scores representing less stress. Cronbach’s alpha = 0.83 within the present sample.

#### Well-Being

Well-being was measured using the 14-item Warwick-Edinburgh Mental Well-being Scale (WEMWBS) a validated and reliable instrument used to measure both hedonic (e.g., happiness and life satisfaction), social (e.g., relationship), and eudemonic (e.g., self-actualization) components of well-being through a unidimensional factor structure (Tennant et al., 2007). Items were anchored on a 5-point Likert scale ranging from “none of the time” (1), to “all of the time” (5). Higher scores indicate better well-being. Cronbach’s alpha = 0.90.

### Data Management and Analyses

#### Data Management

The Statistical Package for Social Sciences (SPSS, version 24) was used for all analyses. On each independent scale, Little’s Missing Completely at Random (MCAR; Little, 1988) was used to assess if responses were missing in random order. Analyses revealed that the data were missing at random (*p* > 0.05), warranting use of the Expectation Maximisation (EM) algorithm for estimating missing values. EM was conducted on each individual scale, using inter-correlated items as predictors, which assumes a logical theoretical structure within the items (Field, 2013).

#### Data Analyses

Descriptive percentage statistics were calculated for gender (i.e., male or female), sport type and sessions completed by the intervention group. Mean and standard deviation scores were for each study outcome at their corresponding time points (baseline and two-weeks follow-up), categorized by intervention and control group. To ascertain if the intervention and control groups differed on any of the demographic variables or study outcomes at baseline, a series of independent samples *t*-tests, and a chi-square test (i.e., for gender) were conducted, with alpha significance set to *p* < 0.05.

All outcome variables were standardized as *z*-scores and difference scores were calculated by subtracting baseline scores from the post-intervention scores. Skewness values ranged from −0.217 to 0.980, while kurtosis values ranged from 0.355 to 1.67. Multicollinearity was not present as all variance inflation factors were below 1.17, and the variables were thus deemed acceptable for regression analyses. All variables were imputed into Hayes (2017) PROCESS macro for SPSS to test the study hypotheses (see section “Study Hypotheses and Models Tested”). In Model 1 (see Figure 1), the intervention (*X*; intervention group coded as 1; control coded as 0) was regressed onto the mindfulness (*M1*), competence (*M2*), stress (*M3*), and well-being (*Y*) difference scores, whereas in Model 2 (see Figure 1), competence replaced mindfulness as *M1*. Gender and age were regressed onto the dependent variable as covariates in both models. Effects on the dependent variable and mediators were inspected through the singular and serial pathways indicated in the study hypotheses. Given the scoring format of the PSS, each of the relationships predicting stress were assumed to be negative.

Model 6 was used in Hayes’ (2017) PROCESS Macro, wherein the effect of *X* on *Y*, and the effect of *X* on the Mediators (i.e., *M1, M2, M3*), was determined through a number of statistical criterion: (i) non-significant (i.e., no relationship); (ii) direct with non-mediation (i.e., mediators do not exert an influence on the relationship); (iii) full mediation (i.e., direct effect is not significant when controlling for mediators’ effect); (iv) partial mediation (i.e., direct effect is significant even when controlling for mediators’ effects) or, (v) indirect (i.e., no direct effect, but *X* exerts an indirect effect on *M2, M3* and *Y* when in sequence with mediators; Hayes, 2009). All effects were examined using a bootstrapping technique, with 10000 samples (Byrne, 2001). Effects were determined statistically significant if confidence intervals did not cross zero (Field, 2013; Hayes, 2017). Completely standardized beta (β) coefficient values were used to assess relationships attributable to the intervention. Moreover, *R*^2^ values were included for the total variance predicted in the model on the difference scores.

## Results

### Participant Demographic and Baseline Analysis

Two hundred and thirty eight student-athletes took part, with 108 in the intervention group, and 130 in the control group. The most commonly reported sports the athletes participated in included: Gaelic Football and Hurling (42%), Soccer (22.5%), Rugby (5.8%), Hockey (5.1%), Basketball (3.6%), Netball (2.9%), and others (18.1%; e.g., Athletics, Combat sports). The mean age of the sample was 20.47 years (*SD* = 3.30), 57.6% were males and 42.4% were females. Regarding demographic differences, the chi-square test revealed that there were no significant gender differences between intervention and control groups (*p* > 0.05). However, the control group had a significantly higher (*p* < 0.05) mean average age (21.39, *SD* = 3.97) than the intervention group (19.45, *SD* = 1.76).

At baseline, a series of independent samples *t*-tests revealed that the intervention and controlled groups did not significantly differ on any of the study outcomes (all *p* > 0.05). Descriptive statistics for the study outcomes are presented in Table 1, showing mean scores for each scale at each time-point, categorized by either intervention or control groups. With regard to adherence, on average the intervention group participated in 3.70 (*SD* = 2.78) mindfulness sessions, with 21.90% reporting engagement with one session, 12.38% at two sessions, and 11.43% at three sessions. Less than 2% of the intervention group reported completing the full available 14 sessions.

**TABLE 1:** Mean scores and standard deviations for scales, categorized for intervention and control participants at baseline and follow-up timepoints.

***Variables***	***Intervention M (SD)***	***Control M (SD)***
*Mindfulness awareness Baseline Two-week follow-up*	54.11 (13.01) 60.02 (9.82)	55.58 (10.90) 60.36 (5.77)
*Competence satisfaction Baseline Two-week follow-up*	21.21 (5.11) 23.21 (3.00)	22.97 (4.60) 23.29 (1.49)
*Stress Baseline Two-weeks follow-up*	18.26 (5.39) 16.75 (3.58)	18.07 (5.96) 16.50 (2.26)
*Well-being Baseline Two-week follow-up*	43.74 (7.20) 47.36 (5.60)	45.41 (8.14) 47.86 (3.89)

**TABLE 2:** Core content of the mental health workshop and mindfulness application.

**Intervention component**	**Key themes**	**Tasks**
*Mental health workshop part 1*	Introduction to mental health concepts (i.e., stress, mindfulness)	Group-based and participant-led discussions on positive framing of mental health and mindfulness as a tool.
*Mental health workshop part 2*	Instructions and how to use the mindfulness application.	One-minute taster meditation, download and try-out of the application.
*Home-directed mindfulness program*	Non-judgmental awareness of the present moment	Guided practices including counting breaths, body scanning and noting thoughts and feelings.

### Main Results

#### Model 1

Results of Model 1 confirmed that in comparison to the control group, the intervention did not significantly enhance changes in the primary mediator of mindfulness (H1). Moreover, the intervention did not indirectly effect changes in competence satisfaction difference scores through mindfulness (H2), or stress through singular (i.e., mindfulness) or double (i.e., mindfulness > stress) sequential pathways (H3 and H4). Lastly, the intervention did not indirectly effect changes in well-being through any of the specified singular, double, or triple mediating pathways tested (H5). Overall, despite the mindfulness practices inherent within the program, the intervention did not exert any direct changes on mindfulness. Further, as the primary mediator, mindfulness did not exert any indirect effects on competence, stress or well-being difference scores. Lastly, as covariates gender and age did not significantly predict well-being.

#### Model 2

**FIGURE 2:**
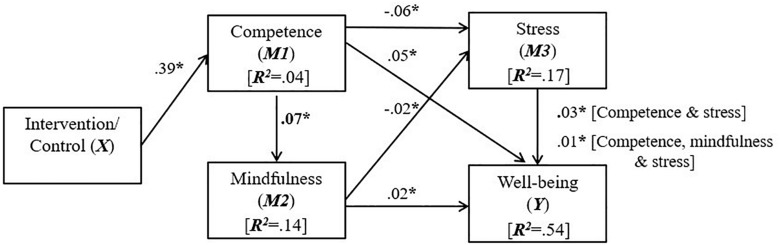
Model 2 showing direct and indirect effects of the mindfulness-based mobile application (*X*) on competence satisfaction (*M1*), mindfulness (*M2*), stress (*M3*), and well-being (*Y*). For visual clarity only significant paths attributable to the intervention were included; ^*^p < 0.05.

When replacing mindfulness with competence as the primary mediator (*M1*) in Model 2, analyses revealed support for H1 such that, in comparison to the control group, the intervention predicted a direct effect on changes in competence satisfaction difference scores (H1; β = 0.39, 95% CI’s = 0.13 to –0.64, *p* < 0.05). Further support was revealed for H2, H3, and H4, to the extent that indirect effects were found for the intervention on mindfulness through competence satisfaction (H2; β = 0.07, 95% CI’s = 0.03 to –0.13, *p* < 0.05); on stress through competence satisfaction (H3; β = −0.06, 95% CI’s = −0.11 to −0.02, *p* < 0.05), and; on stress through competence satisfaction and mindfulness in sequence (H4; β = −0.02, 95% CI’s = −0.04 to −0.00, *p* < 0.05). In respect of H5, the intervention indirectly effected changes in well-being difference scores through competence satisfaction (β = 0.05, 95% CI’s = 0.02 to 0.10, *p* < 0.05); through competence satisfaction and mindfulness in sequence (β = 0.02, 95% CI’s = 0.01 to 0.04, *p* < 0.05); through competence satisfaction and stress in sequence (β = 0.03, 95% CI’s = 0.01 to 0.06, *p* < 0.05), and; through competence satisfaction, mindfulness, and stress in sequence (β = 0.01, 95% CI’s = 0.00 to 0.002, *p* < 0.05). Factoring in all of the variables in the models resulted in a significant proportion of variance predicted for changes in well-being difference scores (*R*^2^ = 0.54), in addition to stress (*R*^2^ = 0.17), mindfulness, (*R*^2^ = 0.14) and competence (*R*^2^ = 0.04). Similar to Model 1, as covariates gender and age did not significantly predict the dependent variable well-being. See Figure 2 for a visual description of Model 2, including significant beta coefficient values.

## Discussion

This study was in response to calls that mental health awareness interventions should be theory-based and when requested be available to student athletes to effectively manage academic, social and sporting stressors (Breslin et al., 2017; Moreland et al., 2018; Shannon et al., 2019). In Model 1, the intervention did not directly affect the primary mediator of mindfulness, exerting no indirect effects on the study outcomes. However in Model 2, the intervention was effective at directly improving changes in competence satisfaction (H1), which subsequently resulted in indirect effects on mindfulness (H2), stress (H3 and H4) and well-being (H5), through SDT mechanisms reflective of competence satisfaction (Ryan and Deci, 2017).

Thus, despite the inclusion of mindfulness practices in the program, the intervention was not effective at directly increasing mindfulness itself, and the positive effects on mindfulness, stress and well-being were all indirectly realized through competence satisfaction (see Figure 2 above). Overall, results indicate that while the enhancement of mindfulness itself does carry some of the responsibility for mindfulness interventions’ effects, as evidenced in indirect effects in H4 and H5 (Brown et al., 2007), the act of engaging with mindfulness training can also foster perceptions of competence in mental health self-management, which may be beneficial to stress regulation and well-being promotion (Ryan and Deci, 2017). However, effect sizes were generally small, and as such, we now discuss findings with a view of advancing mental health interventions for the athlete population.

### Model 1

The intervention’s effects were modeled through SDT using both mindfulness and competence satisfaction as primary and secondary mediators. This approach enabled an empirical inquiry into the mechanisms of change in the program, specifically the temporal nature of the relationship between competence and mindfulness, and their salutary effects, which is considered a crucial step in developing a theoretical underpinning for mental health promotion through mindfulness (Sedlmeier et al., 2018). In Model 1, there was a lack of support for a direct intervention effect on mindfulness changes scores (H1), as measured by the MAAS (Brown and Ryan, 2003). It is likely that the relatively low combined engagement with the program (i.e., on average the intervention participants completed 3.70 sessions) and short duration of the intervention (i.e., 2 weeks) and sessions (i.e., session durations ranged from an optional five through to 20 min) was not a sufficient enough dose to exert direct changes on one’s daily awareness (Creswell, 2017). Indeed, a meta-analyses of 72 mindfulness-based interventions (Visted et al., 2015) reported that approximately 50% have not reported a significant increase in self-reported mindfulness. While some evidence indicates that improvement in mindfulness skills (e.g., counting accuracy of breaths during tasks) are possible during short interventions (Rosenkranz et al., 2019), authors (Cayoun, 2011; Creswell, 2017) have proposed that better adherence to mindfulness programs predicts one’s capacity to achieve heightened mindful states.

To this end, the present intervention may benefit from formative sustainability research that accounts for contextual factors such as the service structure (i.e., how and when the intervention is delivered) and population characteristics (i.e., whether athletes scoring low on well-being may require a longer intervention) (Shelton et al., 2018). Also from a program fidelity perspective, it would have been useful to know the precise amount of time the participants spent meditating (i.e., session length was optional), and moreover, it is possible that the present intervention had effects on additional dimensions of mindfulness not measured by the MAAS. These include non-judgmental reflection and reaction, observation, and descriptions of current experiences, which are assessed in the Five-Factor Mindfulness Questionnaire (FFMQ; Baer et al., 2006). Despite the null findings in Model 1, there were indirect effects present in Model 2, which are of theoretical and practical value to mental health interventions.

### Model 2 Theoretical Implications

Specifically, the finding that improvements in mindfulness difference scores were indirectly predicted by the intervention’s direct effect on competence satisfaction (H1), suggests that the act of engaging with mindfulness practices and instructions can facilitate improved perceptions of competence in mental health self-management, which in turn, produces the conditions that enable one to be mindful and focus on the present (Olafsen, 2017). While SDT hypotheses (Brown et al., 2007) and extant studies (Chang et al., 2015, 2018; Schultz et al., 2015; Campbell et al., 2016; Campbell et al., 2017) propose that the mindfulness construct may precede competence satisfaction, the effects present in Model 2 show support to the contrary. Yet, it should be noted that the null direct effect of the intervention on mindfulness precludes our ability to examine the precise temporal nature of this relationship. Indeed, it may be that there is a bi-directional association between mindfulness and competence satisfaction.

The indirect effects found for the intervention on reducing stress difference scores through competence satisfaction (H3), and competence satisfaction and mindfulness in sequence (H4), supports evidence that mindfulness-based interventions can reduce stress through self-regulatory mechanisms (Gu et al., 2015; Vidic et al., 2017). Specifically findings are theoretically aligned with a SDT perspective (Ryan and Deci, 2017) that the manner in which one appraises and is aware of mental health challenges is crucial, to the extent that improved competence satisfaction can result in better self-regulation of environmental stressors (Weinstein and Ryan, 2011). Indeed, research studies have shown that competence independently predicts reduced stress and improved well-being (Jex et al., 2001; Mikolajczak et al., 2015), and the present intervention’s effect support, respectively, a positive direct, and inverse indirect, relationship with competence and stress through mindfulness-based programs. Given student-athletes experience multiple social, academic and sporting stressors, and often report a lack of ability in self-regulating stressors (Moreland et al., 2018), improving competence through provision of mindfulness training may be of value. Such efforts may be aided by longer-lasting interventions that provide sufficient time to directly improve mindfulness skills (Cayoun, 2011), and from a theoretical perspective, may help disentangle the temporal relationship between competence satisfaction and mindfulness.

Supporting H5, competence, mindfulness and stress indirectly predicted the interventions positive effects on well-being difference scores, as measured by the WEMWBS (Tennant et al., 2007). The specific skills taught to the participants through the mindfulness program, including improving awareness of the concept of mindfulness, and the relationship between thoughts, breathing and attention, may have improved student-athlete’s well-being through the key medium of competence needs satisfaction (Brown and Ryan, 2003; Weinstein and Ryan, 2011). It is well acknowledged that competence satisfaction is robustly related to improved well-being (Ryan and Deci, 2017), however, this is the first methodologically rigorous mindfulness-based study to test such indirect mechanisms through statistical mediation analyses among athletes.

Mindfulness and stress both accompanied the intervention’s indirect effects on well-being through competence, supporting the view that mindfulness-based programs can help individuals feel effective at self-regulating the stressors that are predictive of mental health (Creswell, 2017). Indeed, inclusion of the mediators alongside the intervention and control group resulted in a significant proportion of variance explained for well-being difference scores in the model (*R*^2^ = 0.54). What was not considered in model 2, was the intervention’s effects on distinct eudemonic, hedonic and social well-being constructs (Keyes, 2005), and additional mental health domain-specific measures of autonomy and relatedness. Although the WEMWBS (Tennant et al., 2007) items do tap into such components, its unidimensional structure permits the examination of precise pathways. Hence, it may be worth including multi-dimensional mental well-being measures in future studies, such as the mental health continuum (Keyes, 2002) that has recently been applied to mental health in sport (Uphill et al., 2016), and further mental health domain measures of autonomy and relatedness.

### Generalizability and Limitations

The key contribution of this study was the inclusion of SDT to test the mechanisms of change in a mindfulness-based mental health intervention among athletes. While this research showed support for indirect mechanisms which are of theoretical and practical value (Creswell, 2017), effect sizes were generally small, and the study is also not without its limitations. These include: the lack of a long-term follow-up period which prevents determining whether effects extended beyond two-weeks; a lack of randomization to groups; a relatively small level of adherence to the full mindfulness program, and; a full testing of SDT components (i.e., autonomy and relatedness satisfaction for mental health). A further and longer-lasting SDT-based mindfulness intervention is warranted that accounts for these limitations. From a practical standpoint, researchers have suggested better adherence to mental health interventions when athletes feel the program is aligned, and sensitive to the nuances of sports performance culture (Gavrilova et al., 2017). Such examples do exist, such the Mindfulness-Acceptance-Commitment Program (MACP; Gardner and Moore, 2004), which has been linked to both positive sporting and mental health outcomes (Gardner and Moore, 2007; Gross et al., 2016; Zhang et al., 2016), and may be aided by application of SDT and online modalities. When utilizing such approaches, researchers and practitioners may be cautious of the remaining open-questions regarding potential risks of online mindfulness interventions, in addition to financial and technological barriers (Creswell, 2017). From a measurement perspective, further research could apply multicomponent measures of mindfulness (see, Baer et al., 2006) and develop mental health domain-specific autonomy and relatedness scales. In this vein, researchers may consider good practice in psychometrics (Hagger and Chatzisarantis, 2009). Further interventions may also consider program fidelity aspects, such as the length, duration and participant adherence to the mindfulness sessions, in addition to training deliverers in SDT principles, as conducted in the present study (see Shannon et al., 2018 for an example of needs-supportive teacher training). Various level of sport participation among the athletes (e.g., elite, semi-elite, amateur), current mental health levels (e.g., flourishing, moderate or languishing mental health, see Keyes, 2002), and past participant experience in mental health self-management training (e.g., CBT) may also be considered.

## Conclusion

Psychological well-being is facilitated by an awareness of and ability to self-regulate stressors (Weinstein and Ryan, 2011). As student-athletes frequently report the presence of multifaceted sporting, academic and social stressors (Moreland et al., 2018), the present study sought to examine the efficacy of a mental health intervention for reducing stress and promoting well-being, whilst also contributing to theoretical understanding of the mechanisms of change in mindfulness interventions. Support was found for the competence-promoting processes in the intervention, to the extent that the act of engaging with mindfulness practices can foster perceptions of competence in mental health self-management, which exerted indirect intervention effects on mindfulness, stress regulation, and ultimately, psychological well-being. Overall, we propose that mindfulness-based mental health interventions may offer a way to promote mental health among athletes, with the caveat that attention is given to the promotion of competence in such programs. However, effects were generally small, and there are a number of remaining theoretical and practical questions to addressed. Specifically, as the present intervention was not effective at directly increasing mindfulness, the temporal association between competence satisfaction and mindfulness, and their salutary effects, remains open for further assessment. From a practical viewpoint, we suggest that longer-lasting programs tailored for sports culture are warranted (Gavrilova et al., 2017), in which those involved in program design control for intervention accessibility and sustainability, adherence, duration and intensity of mindfulness sessions, in addition to theoretical application by deliverers and potential risks (Creswell, 2017). Moreover, future programs may consider theoretically driven mindfulness interventions in all aspects of design and analyses that are conducted through a longitudinal experimental design, in which allocation to groups is randomized. To conclude, a mindfulness-based mental health intervention was associated with reduced stress, and improved well-being among athletes through SDT mechanisms reflective of competence satisfaction.

## Data Availability

The datasets for this study are available under reasonable private request, however, we do not have ethical permission for public sharing. Requests to access the datasets should be directed to g.breslin@ulster.ac.uk.

## Ethics Statement

This study was carried out in accordance with Ulster University ethical guidance. All subjects gave written informed consent in accordance with the Declaration of Helsinki. The protocol was approved by the Sport and Exercise Sciences Research Institute filter committee.

## Author Contributions

SS and GB designed the study. SS, TH, and CM collected the data. DN and ML delivered the intervention. SS, DH, and GB analyzed the data. All authors contributed to the final drafting of the manuscript.

## Conflict of Interest Statement

The authors declare that the research was conducted in the absence of any commercial or financial relationships that could be construed as a potential conflict of interest.

## Footnotes

1 Both deliverers were trained in leading the workshops through an autonomy-supportive style, and training was adapted from previous interventions using SDT in the physical activity domain (Shannon et al., 2018).
